# Understanding of research, genetics and genetic research in a rapid ethical assessment in north west Cameroon

**DOI:** 10.1093/inthealth/ihv034

**Published:** 2015-05-13

**Authors:** Jonas A. Kengne-Ouafo, James D. Millard, Theobald M. Nji, William F. Tantoh, Doris N. Nyoh, Nicholas Tendongfor, Peter A. Enyong, Melanie J. Newport, Gail Davey, Samuel Wanji

**Affiliations:** a Department of Microbiology and Parasitology, University of Buea, PO Box 63, Buea, Cameroon; b Wellcome Trust Centre for Global Health Research, Brighton and Sussex Medical School, Falmer Campus, Brighton BN1 9PX, UK; c Research Foundation in Tropical Diseases and Environment, PO Box 474, Buea, Cameroon; d Department of Sociology and Anthropology, University of Buea, PO Box 63, Buea, Cameroon; e Department of Sociology, University of Douala, Cameroon

**Keywords:** Cameroon, Elephantiasis, Podoconiosis, Rapid ethical assessment

## Abstract

**Background:**

There is limited assessment of whether research participants in low-income settings are afforded a full understanding of the meaning of medical research. There may also be particular issues with the understanding of genetic research. We used a rapid ethical assessment methodology to explore perceptions surrounding the meaning of research, genetics and genetic research in north west Cameroon.

**Methods:**

Eleven focus group discussions (including 107 adults) and 72 in-depth interviews were conducted with various stakeholders in two health districts in north west Cameroon between February and April 2012.

**Results:**

Most participants appreciated the role of research in generating knowledge and identified a difference between research and healthcare but gave varied explanations as to this difference. Most participants' understanding of genetics was limited to concepts of hereditary, with potential benefits limited to the level of the individual or family. Explanations based on supernatural beliefs were identified as a special issue but participants tended not to identify any other special risks with genetic research.

**Conclusion:**

We demonstrated a variable level of understanding of research, genetics and genetic research, with implications for those carrying out genetic research in this and other low resource settings. Our study highlights the utility of rapid ethical assessment prior to complex or sensitive research.

## Introduction

Globally, a disproportionate burden of disease falls upon low-income countries,^1,2^ yet medical research in these settings has suffered relative neglect. Low-income countries are therefore particularly likely to benefit from well conducted, appropriate medical research.^3^ However, externally sponsored research may raise several issues of concern and in recent years there has been increasing interest in the ethical challenges of research in these settings.^3–6^ Despite this, relatively little has been done to assess whether participants in medical research and members of their community are afforded a full understanding of what medical research is, what its underlying principles are and what it means to them.

Medical researchers are likely to accept as implicit certain central tenets of research methodology and its distinction from routine health care. Research participants and their communities may lack this research paradigm, may be unfamiliar with processes common in medical research and be more likely to frame their understanding of research within a routine health care paradigm.^7–13^ In particular, concepts that appear to be uniquely difficult to comprehend include those held as most central by researchers; clinical equipoise, placebo, randomisation and blinding.^7,14–18^ Poor understanding of these concepts may co-exist with very good understanding of the procedural aspects of research (e.g. which tablets to take and when) and thus be difficult to appreciate.^6,14^ Much has also been written regarding the so called ‘therapeutic misconception’ amongst participants in medical research. First described in 1982,^19^ the therapeutic misconception refers to the belief that participation in medical research will be of direct clinical benefit to the participant rather than simply of potential, future benefit to others.^7,8,18,20–22^

There has been a large increase in genetic and genomic research, to the extent that even research for which the primary focus is elsewhere often includes some degree of genetic research.^23–25^ Genetic research may have special implications for participants, for instance specimens may be stored and consent given for future, unknown studies and release of genetic information could have implications for insurance, employment or lead to discrimination or damage to family relationships.^24,25^ In addition, participants do not usually derive any personal benefit from the research and thus the issue of the ‘therapeutic misconception’ may be particularly acute.^24,26^ These issues are compounded by the fact that genetic concepts may be particularly difficult to comprehend.^6,24–26^ There remains a need for a more nuanced understanding of what research participants and their communities understand by medical research in general and genetic research in particular.

Rapid ethical assessment is a qualitative technique that aims to assess ethical issues particular to a given region and particular to a given proposed research topic, before research begins, in order to better achieve attainment of universal research principles in a specific research context.^27^ This approach has been used in The Gambia and then subsequently in Ethiopia prior to a study to investigate the genetic basis of podoconiosis.^21,27,28^ Similarly, in this study we performed a rapid ethical assessment before a study of the genetic basis of podoconiosis and used comparable techniques utilising focus group discussions (FGDs) and in-depth interviews (IDIs), again involving both community members and health professionals.

In this article, we describe perceptions surrounding the meaning of research, genetics and genetic research. We believe that this study will be useful to future researchers planning biomedical research, particularly genetic research in Cameroon and further afield. Moreover, there is increasing evidence of the relevance and acceptability of the rapid ethical assessment methodology to researchers in low-income settings and we wished to further explore its applicability.^29^

## Methods

### Study area

Two areas corresponding to the Tubah and Ndop health districts, in the Tubah and Ngoketunjia sub-divisions respectively, in north west Cameroon were selected. Twenty-two health areas were visited (nine in Tubah and 13 in Ndop). Podoconiosis has been identified in both districts.^30^

### Study design

IDIs and FGDs were conducted prior to the planned podoconiosis genetic research, between February and April 2012 using a methodology previously described^31^ and building on similar methods used in The Gambia and Ethiopia.^21,27^ IDIs and FGDs were both used in order to explore different but complimentary areas; FGDs allow further understanding of how perceptions of individual issues are altered within community groups, IDIs allow exploration of individuals' experiences, perceptions and feelings around potentially sensitive and stigmatising issues, an area of active concern in podoconiosis.^32–34^ A purposive sampling technique was used for the recruitment of health workers, NGO members and community leaders, while a random sampling technique, stratified by age and gender, was used for the recruitment of community members. Recruitment continued until analysis identified no new themes.

### Study participants

Seventy-two IDIs and 11 FGDs, including six all-male and five all-female FGDs, with a total of 107 participants were conducted. Forty-five of the IDIs were conducted in Ndop (38 with male and seven with female participants) and 27 in Tubah (20 with male and seven with female participants). IDI participants in both districts were grouped into four categories; 1) health workers (n=12), 2) NGO leaders (n=9) and NGO members (n=11), 3) community members (n=23), community leaders (n=14), traditional ruler (n=1) and local government officer (n=1), and 4) research scientist (n=1). FGDs comprised 30 ‘young’ (18–25 years) and 20 ‘adult’ (>25 years) male participants as well as 24 ‘young’ and 33 ‘adult’ female participants. FGD group size ranged from 6–12 individuals. IDIs all lasted for 20 to 30 minutes, FGDs lasted from 25 to 50 minutes.

### Data analysis

Data were collected, transcribed anonymously and coded following themes. Data in Pidgin was translated into English prior to transcription. Translation efficiency and codes' development were performed as previously described.^31^ The themes addressed in this paper include understanding of research, understanding of genetics and understanding of genetic research in general. Whilst we did ask questions specific to podoconiosis in the course of this research, these are not reported here.

## Results

A total of 179 participants (70 female and 109 male) aged between 21 and 66 years, took part in the study. Educational attainment differed by group with most community members having no formal education, most fieldworkers and NGO leaders having secondary education and most health workers having higher level education.

### Participants' understanding of research

Participants were asked for their understanding of research. Community members described a process whereby new knowledge is obtained by asking, studying, observing or performing trials:

*Research is a process through which observations and investigations are made in order to discover, to find out something*. (male community member, IDI, Tubah).

*Research is taking a particular problem and studying how it came about*. (female community member, IDI, Ndop).

Familiarity with research was shown through exposure to agricultural research, and perhaps as a consequence was articulated using farming illustrations:

*Research is like trying something to see whether it can work. This is the way I understand it as a lay man. For example if you can plant corn on stones, it does not grow and you plant the same corn on fertile land and it grows then you are doing research.* (male community member, IDI, Ndop).

In addition to the assimilation of knowledge, participants suggested that research leads to improvement or change on the basis of this knowledge:

*Research is when people go out to check whether things are good and can help the people* (community leader, IDI, Ndop).

Health workers and NGO leaders had a conception of what research was. Similarly to the community members they described a process leading to increased knowledge:

*When they talk about a research study, they mean they want to carry out a study on something to discover it. For example they may want to carry out a study on HIV, maybe to discover the drug or how to cure people or to know how to prevent it.* (health worker, IDI, Tubah).

However, health workers and NGO leaders tended to have a broader conception of what research might entail and to be more explicit in their conception of research as a scientific endeavour:

*Research can be seen as the scientific study of some diseases in the health domain*. (NGO leader, IDI, Ndop).

They were also more specific in detailing elements of the process leading to increased knowledge, for instance outlining analysis, rather than simply the collection of, data:

*Research is to study a particular thing to see whether it is there and what can be done. For example if it concerns health, you have to study how frequent it is. It is also to know whether people are aware of and how the research team can help the situation. To do that, they collect data and analyse it and give the feedback. They are trained for that*. (health worker, IDI, Ndop).

*Research–the collection of information on a particular subject for analysis*. (NGO leader IDI, Tubah).

In general they were also more specific when reporting a role for research in the development of new interventions, particularly mentioning pharmaceutical interventions:

*Research is a way to find the reason why something exists. Research is to evaluate, to find out the causes of a disease and discover drugs that can be used to cure that disease*. (health worker, IDI, Ndop).

Overall, community members appeared to have views which, although expressed differently from one another, indicated a sound conceptual understanding of what research might be. Likewise, health workers and NGO leaders had good, but usually more nuanced understanding of research.

### Health workers' and NGO leaders' views of community understanding of research

Health workers and NGO leaders were asked how well they thought ‘their’ community members understood research. Previous research exposure was held to be important in the understanding of research:

*I think so, because researchers come here very often for different research work and as such they should know and understand much about it*. (Field worker, IDI, Tubah).

However, doubts were expressed as to whether the community would understand the concept of research:

*No, not quite, this is a rural area; only a few people will understand the meaning of research*. (NGO leader and health worker, IDI, Tubah).

### Community participation in research

It was felt that community understanding of research may be materially altered by whether they had previously taken part in research. Most community members reported having taken part in (primarily agricultural) research at least once. It should be noted however, that some groups (particularly women) felt excluded from research:

*Many people know we don't know anything so they go to men all the time.* (adult female, FGD, Ndop).

Some community members had never taken part in any research before. Research-naïve individuals were found in all groups and both districts:

*No I have never participated in any research before*. (male community member, IDI, Tubah).

### Understanding the difference between research and health care

Importantly we found understanding of research amongst community members expressed in terms closer to those used for health care:

*I think research is all about improving on the quality of life that is making us to be healthy and not allowing people to die as if they were suffering*. (female community member, IDI, Tubah).

This issue was explored in more depth, in order to understand the extent to which participants could distinguish between research and health care. A range of conceptions were found; with community members considering there to be a clear difference between research and health care, but not expanding fully on what the difference might be:

*Research and health care are not the same. With health care one does not go to the field. These are two different things*. (male community member, IDI, Tubah).

Alternatively, participants reported a clear difference and described what health care might look like, but did not expand on what research might be in contrast:

*Yes, there is a difference between research and health care, which is taking care of sick people*. (community leader, IDI, Ndop).

*Research is different from health care, which is to take care of your body*. (community leader, IDI, Tubah).

Several participants felt there was a clear distinction between research and health care and were able to articulate and compare the two concepts:

*Truly, we know that there is a clear difference between research and treatment; we know that with research you people are to find out the reason we are suffering from some sickness and meanwhile treatment is giving us (medicine) for the sickness that is disturbing us*. (adult male, FGD, Tubah).

In contrast, another conception was to believe that there was no difference between research and health care, but to see them as part of the same overall process:

*Research and health care are the same*. (female community member, IDI, Ndop).

A theme within this group was to conceive of research as being to do with diagnosis and health care as being to do with treatment:

*Health care and research are the same because you start with research before you give drugs in the hospital*. (community leader, IDI, Ndop).

*I am not sure there is a difference, for me they are the same because if you don't examine a patient you cannot treat him or her*. (male community member, IDI, Tubah).

It is notable that in this instance the participants refer to both health care and research at the individual level, whereas most research is unlikely to directly benefit an individual's diagnosis in this way. Apart from some of these individuals who considered research and health care to be part of the same process, participants did not generally believe research to have immediate therapeutic benefit.

### Participants' understanding of genetic research

Generally ‘genetics’ was not a term familiar to community members. However, after a basic explanation (briefly highlighting the role of genes, heritability and variation between related individuals), members were able to demonstrate some pre-existing understanding of concepts, if not the term itself. Understanding was demonstrated by describing hereditability rather than other aspects of genetics:

*Genetics means studying blood relationship*. (adult male community member, FGD, Ndop).

*Genetics is something that moves within the family blood for example the albino gene*. (adult male community member, FGD, Ndop).

Similarly, when asked whether genetic studies were important to them, respondents stressed the possibility of identifying causes or interventions for hereditary diseases:

*Genetic studies are important because from these studies we could discover an illness that is in that family*. (adult male community member, FGD, Tubah).

*The study is important because illnesses can be traced in families and treated or terminated*. (community leader, IDI, Ndop).

It may be noted that in the comments above, community members appear to allude to benefits from genetic research at the level of the individual or family. No participants discussed the relevance of genetic research in terms of benefit to the community or wider group.

### Issues specific to genetic research

Participants in both health districts were of the view that genetic research did not pose any special problems or risks not encountered in other research settings:

*No, I don't see any problem*. (health worker, IDI, Tubah).

*No, it does not pose any problem*. (health worker, IDI, Ndop).

*No special problem at all*. (health worker, IDI, Ndop).

Of course, if participants are unaware of the nature of genetic research and key differences from other research, it can reasonably be doubted that they would identify any unique issues.

An issue discussed by health workers which appeared particular to genetic studies was the potential to consider genetics to be synonymous with heredity and thus stigmatising, as well as explanations based on the supernatural:

*They don't understand easily because sometimes they think that the family is cursed. Their illness is due to witchcraft. Those who are educated understand that some diseases are hereditary belonging to a family*. (health worker, IDI, Tubah).

*For that you really need a lot of training, a lot of information for people to understand genetics for many people believe in witchcraft*. (health worker, IDI, Tubah).

## Discussion

Despite increased focus on the issues posed by research in resource limited settings,^3–8,14,17,22^ little, if any, research has assessed the core understanding of what research is amongst communities in these settings. We have presented the results of a rapid ethical assessment performed in north west Cameroon which was designed, amongst other things, to assess understanding of research in general and genetic research in particular, in order to inform a study into the genetic basis of podoconiosis.

Concerns have been raised about individuals' ability to understand the concept of research in resource limited settings, where educational attainment is often low.^5,7,14,18,27^ We found a more complex picture whereby participants were generally able to give an accurate description of the principal aim of research, that of increasing knowledge by various methods. This finding is similar to qualitative research from Malawi^35^ and Sri Lanka^36^ where participants were generally able to describe this fundamental role of research with alternative descriptive words. Some community members appeared to derive their understanding of research from exposure to agricultural research and most reported having been approached to participate in research of one type or another. Health workers and NGO leaders also felt that community members' knowledge of research would be good as a consequence of previous exposure. Similarly, in the Malawian and Sri Lankan settings many participants had participated in previous research.^35,36^ However, this generally reasonable understanding of the concept of research may not hold in all resource limited settings, particularly those without prior exposure to research.

Several community members then went on to suggest that knowledge from research may be used for improvements in the future. However, only health workers and NGO leaders referred to specific research methodologies and specific examples as to how research findings might be used. It is likely, but not proven, that differences in educational attainment and occupational experience may have provided this more in-depth understanding in the case of health workers and NGO leaders.

We aimed to explore participants' understanding of the difference between research and health care. A minority of participants felt there was no difference at all. Most participants felt there was a difference, however, there were varying levels of ability to explain what this difference might be. In particular, those who felt that there was a difference between the two appeared to have greater difficulty explaining what research was than explaining what health care was. Also, some of these participants described research in a manner that would more usually be thought of as diagnostic investigations within healthcare. This was similar to the findings from the studies in Malawi and Sri Lanka,^35,36^ and gives us further insight into what may drive the ‘therapeutic misconception’.^19^ If research is understood to be a part of health care rather than separate to it, then from the perspective of the potential research participant it may seem reasonable to assume that something new and better is being offered. However, in contrast to concerns previously raised over the importance of the ‘therapeutic misconception’ and the results from the Malawian study,^35^ participants who felt that there would be an immediate clinical benefit to research participation were in a distinct minority in this study. Again, this pattern may not be generalizable to other resource poor settings and may reflect this particular community with a given level of previous research sensitization.

We also explored participants' understanding of genetics. Few community members had an understanding of the term genetics itself. However, given a brief explanation of the term, many were able to go on and demonstrate some knowledge of genetic concepts. It was striking that the majority of participants with some understanding of genetics referred to hereditary disease. No participant described genetic concepts that could be understood to give an appreciation of, for instance, DNA, the role of genetic polymorphisms or genome-wide association studies. This is similar to the findings of a qualitative study amongst participants in the MalariaGEN study of genetic factors influencing immune responses to malaria in northern rural Ghana.^26^ Understanding ‘genetic’ as synonymous with ‘hereditary’ for many people in this setting is perhaps understandable as hereditary diseases may be most ‘visible’ (in contrast to other genetic concepts) and thus correlate best with existing understanding. These points are important to appreciate because an increasing amount of genetic research in resource poor settings is likely to be of this type (e.g. genome-wide association studies of complex diseases), rather than studies of directly hereditable disease. Understanding ‘genetic’ as synonymous with ‘heritable’ may also lead to unhelpful assumptions about determinism. In a prior study examining the link between beliefs about hereditary and behaviours in relation to podoconiosis in Ethiopia, a degree of determinism was noted with respect to this disease, which was sometimes held to be hereditary (as opposed to an environmental disease with genetic predisposition, as is actually the case) and thus inevitable.^37^ It may be the case that if genetic research is felt to be solely concerned with simple Mendelian disease and if there is no understanding that genetic susceptibility may be modulated by environmental exposure, this may affect understanding of and recruitment to genetic studies.

Misconceptions of what ‘genetic’ means may lead on to several specific issues for genetic research which were identified in our study. The first of these, identified by several health workers and NGO leaders, is that the hereditary diseases were, in their opinion, often felt to be due to witchcraft. This may have significant implications for those tasked with consenting and recruiting participants for genetic research and may represent a barrier to recruitment. Secondly, when asked what the relevance of genetic studies might be, participants were almost universal in describing putative benefits at the level of the individual or family. This makes sense if the understanding of genetics is limited to hereditary. This is important because, for instance, genome-wide association studies are unlikely to have any direct benefit to an individual participant or their family. This links again to the idea of the ‘therapeutic misconception’ and has implications for informed consent to genetic studies. Most participants felt that there were no other special issues raised by the nature of research involving genetics as opposed to research in general, whereas in point of fact, a number of issues particular to genetic research exist. The fact that no special issues were raised as a concern may relate to the more limited understanding of genetic research detailed above. This finding is similar to that from the Ghanian study, where good understanding of the procedure of the trial was in contrast to poorer understanding of the special implications of genetic research.^26^

### Limitations

There are several limitations to this study. Whilst women were well represented within the FGDs, they were under represented in the IDIs, largely as a function of community leaders, health workers and NGO leaders being more likely to be men. Interviews were conducted in both English and Pidgin, whereas analysis was performed in English which may have masked some nuances in meaning. Rapid ethical assessments are a relatively new methodology, albeit emerging from amongst a wider literature of rapid assessment techniques. This necessarily means that certain general criticisms of the technique also apply to this project. Rapid ethical assessments are targeted at fairly specific questions prior to a specific research study, thus findings are likely to be highly context specific and not necessarily be generalizable to other circumstances.

### Conclusions

In summary, using a rapid ethical assessment, we demonstrated a variable level of understanding of the fundamentals of research in general in north west Cameroon. Previous research exposure may have contributed to this level of understanding. Most participants felt that there was a difference between research and health care but there were varying explanations as to what this difference might be. To some extent the ‘therapeutic misconception’ is therefore likely to influence participation in research in this setting. We have demonstrated a mixed level of understanding of genetics and genetic research in this setting, with most participants' understanding limited to concepts of hereditary and possible benefits seen in individual or family terms. Special risks associated with genetic research tended to not be identified. We were able to use these findings to tailor the information provided during the consent process for the subsequent podoconiosis genetic research to explicitly address these issues. These findings have implications for those carrying out genetic research in this and other low resource settings. Our study also highlights the utility of rapid ethical assessment prior to research in complex or sensitive areas.
